# Circadian typologies and insomnia in individuals with internet gaming disorder comorbid with attention deficit/hyperactivity disorder

**DOI:** 10.1038/s41598-023-39462-2

**Published:** 2023-08-07

**Authors:** Yen Ju Lin, Ju-Yu Yen, Pai-Cheng Lin, Hui-Yuan Liao, Chih-Hung Ko

**Affiliations:** 1 Department of Psychiatry, Kaohsiung Medical University Hospital, Kaohsiung Medical University, Kaohsiung City, 807 Taiwan; 2 Department of Psychiatry, Kaohsiung Municipal Ta-Tung Hospital, Kaohsiung Medical University, Kaohsiung City, 812 Taiwan; 3 Department of Psychiatry, Faculty of Medicine, College of Medicine, Kaohsiung Medical University, Kaohsiung City, 807 Taiwan; 4 Department of Psychiatry, Kaohsiung Municipal Siaogang Hospital, Kaohsiung Medical University, 482, Shanming Rd, Siaogang Dist., Kaohsiung, 812 Taiwan

**Keywords:** Psychology, Diseases, Signs and symptoms

## Abstract

The alteration in circadian typology and insomnia were prevalent among both Individuals with IGD and those with attention deficit hyperactivity disorder (ADHD), the most comorbid psychiatric disorder of IGD. This study aimed to evaluate the relationships between circadian typologies, insomnia, and internet gaming disorder (IGD) and how ADHD affects this relationship. We recruited three groups of 69 young adults: an IGD group, a control group comprising age- and sex-matched nongamers, and a group of gamers without IGD through diagnostic interviews. The participants with IGD exhibited lower composite scale of morningness (CSM) scores and thus a higher eveningness preference In addition, the score of Pittsburgh insomnia rating scale—20-item version (PIRS_20) was significantly higher among those with IGD. The participants with IGD and ADHD exhibited lower CSM scores but higher PRIS_20 scores than the participants with IGD but without ADHD. The present findings indicate that participants with IGD exhibited a tendency of eveningness preference and experienced more severe insomnia. ADHD exacerbated the eveningness preference and insomnia of individuals with IGD. Close attention should be paid to sleep problems in individuals with IGD, particularly to those with ADHD.

## Introduction

Gaming technology has advanced considerably in recent decades, and the Internet becoming accessible to more people has led to Internet gaming disorder (IGD) becoming a global mental health concern. The *Diagnostic and Statistical Manual of Mental Disorders, Fifth Edition* (*DSM-5*) presents criteria for diagnosing IGD^1^ and the principal symptom is stated as being persistent and recurrent engagement in online gaming leading to impairment or distress that has clinical significance. Excessive Internet gaming has been found by scholars to associate with maladaptive gaming beliefs and to have negative psychological consequences, including problems in interpersonal impairment, and depression^2–4^. Because inadequate or nonrestorative sleep can remarkably reduce a person’s quality of life, understanding the association between IGD and sleep is crucial. Research has indicated that IGD is related to low sleep quality, limited sleep time, and subjective insomnia^5–7^. Insufficient experimental evidence is currently available regarding the sleep patterns of individuals with IGD. Therefore, understanding circadian typologies among individuals with IGD is essential.

### Circadian typologies of IGD

The circadian rhythm of individuals is expressed using chronotypes or circadian typologies, which cover a continuum between morningness and eveningness^8^. Individuals’ preferences for the timings of sleep and wakefulness are classified into the morning, intermediate, and evening types. The sleep–wake schedules of individuals with morningness preference are considerably more regular than those of individuals with eveningness preference^9^. Circadian rhythmicity is reportedly associated with addiction^8^. Cross-sectional research has revealed that a significant correlation exists between eveningness preference and increased compulsive Internet usage^10,11^. Research that involved 532 students revealed mobile phone or computer usage before sleep to be negatively and positively correlated with morningness preference and the late chronotype, respectively^12^. Moreover, a 1‐year longitudinal study determined that in children and adolescents, disturbed circadian rhythm was prospectively predicted by Internet addiction^13^. How Internet addiction, electronic media use, and circadian rhythmicity are interconnected has been the subject of much research, but attention has been lacking in regards to the relationship in adults of circadian typology with IGD; thus, research is required on this relationship.

### Insomnia in individuals with IGD

Studies have determined how sleep problems are related to social media use on mobile phones^4,12,14^ and Internet addiction^13,15^. Lam (2014) reported correlations among sleep problems^6^, problematic Internet usage, and online gaming addiction in their systematic review. Further, under objective device assessment, gaming or social media use was associated with low physical activity and low sleep quality^16^. IGD is assumed to be closely related to sleep problems. In numerous cross-sectional studies^4,17^, IGD has been discovered to be positively correlated with sleep disruption and insomnia. In addition, the amount of time a person spends gaming was found to significantly negatively correlate with insomnia, fatigue, rise time, and bedtime in adults^18^. Studies have investigated IGD by using nonstandard approaches (such as questionnaires), and this may have led to functional impairment being defined in a limited manner and IGD severity being overestimated. Therefore, the relationship between IGD and insomnia should be evaluated through diagnostic interviews.

### Relationships of attention deficit hyperactivity disorder with circadian typologies and insomnia in individuals with IGD

Studies have reported the relationship between IGD and attention deficit hyperactivity disorder (ADHD)^19–21^. Gentile^22^ found that those exhibiting pathological video-gaming behaviors during adolescence were at 2.77-times higher risk of an ADHD diagnosis than those who did not exhibit such behaviors. Various models have been developed to describe the ADHD–IGD relationship. A positive correlation was found between IGD and ADHD, particularly for the inattention subscale, in the systematic review conducted by Dullur et al.^19^. Furthermore, sleep and circadian rhythm problems in children and adults with ADHD have attracted research attention. Individuals with ADHD exhibit more frequent circadian and sleep disorders, a delayed circadian rhythm or later chronotype, and a shorter sleep time than those without the disorder^23,24^. One study identified that circadian rhythm and sleep problems may be causatively related to ADHD^25^. Thus, ADHD appears to affect the relationships between circadian typologies, insomnia, and IGD. Consequently, a study examining the effects of ADHD on IGD and sleep patterns is required.

### Aims of this study

The present study had the following two aims: (1) to examine whether circadian typologies, insomnia, and IGD are correlated in three groups: regular gamers, gamers with IGD, and a control group (nongamers); and (2) to investigate the relationships of ADHD with circadian typologies and insomnia among individuals with IGD. We formulated the following hypotheses: (1) circadian typologies and insomnia are associated with IGD; (2) individuals with IGD experience more severe insomnia and exhibit a stronger tendency for eveningness preference than do those without IGD; and (3) ADHD moderates the relationships between circadian typologies, insomnia, and IGD. In contrast to other relevant studies, a group of regular gamers was included. The inclusion of these individuals made it possible to understand the effects of gaming or addiction to gaming on circadian typologies and insomnia.

## Methods

### Participants

From April 2017 to February 2018, we placed advertisements on campuses and university bulletin boards to recruit participants, who were placed in one of three groups on the basis of whether they were gamers and had IGD. These groups were a control group (those who did not game online), an IGD group, and a regular gamer group (those who did game online but did not have IGD). The participants in the three groups were matched in terms of age and sex. For the IGD group, the inclusion criteria were as follows: (1) age of 20–38 years and more than 12 years of education; (2) engaging in ≥ 4 h (weekdays) and ≥ 6 h (weekends) per day of online video game activity; and (3) gaming online in a consistent pattern for more than 2 years. This corresponded to more than half of these participants’ free time being spent on online gaming and this pattern having been exhibited for a long duration. A psychiatrist conducted interviews with those who met these criteria and used the *DSM-5* diagnostic criteria for IGD^1^ determine whether they had IGD.

The screening criteria for the control and regular gamer groups as well as the IGD *DSM-5*-based diagnostic interview process are detailed in Ko et al.^26^. The diagnostic interviews consisted of (1) the Mini International Neuropsychiatric Interview (Chinese version) to ensure that none of the potential participants had bipolar I disorder, substance abuse disorder, or a psychotic disorder and (2) taking of history to ensure that none had an intellectual disability, brain injury, or a severe physical disorder. This study recruited 69 participants per group, meaning 207 in total. The participants had to provide informed consent to be included^26^.

### Measures

#### Diagnosis of adult ADHD

A psychiatrist evaluated all the participants for adult ADHD in accordance with the relevant *DSM-5* criteria. The psychiatrist determined whether the participants had exhibited various ADHD symptoms prior to the age of 12 years. A participant who fulfilled at least five inattention or hyperactivity and impulsivity criteria received a diagnosis of adult ADHD.

#### Composite scale of morningness

The Composite Scale of Morningness (CSM) is a Likert-type scale with 13 items and is widely adopted to determine whether an individual has morningness or eveningness preference^9^. Individuals can be divided into three categories in accordance with their CSM score, which ranges from 13 to 55 (corresponding to extreme eveningness and morningness, respectively): morning type (CSM score ≥ 44), intermediate type (CSM score between 23 and 43); and evening type (CSM score ≤ 22). A higher CSM score indicates a self-perception of an early bedtime and a high level of morning activity. The CSM has a Cronbach’s α value of 0.87^27^ in the original study and 0.84 in this presenting study.

#### Pittsburg insomnia rating scale—20-item version

The original version of the Pittsburg Insomnia Rating Scale comprises 60 self-report items; this study used the 20-item version (PIRS_20), which measures daytime and nighttime distress symptoms (12 items); sleep parameters (4 items); and sleep depth, quality, and regularity (4 items) in the preceding week^28,29^. Each item in the PIRS_20 is scored on a 4-point Likert scale ranging from 0 (*not at all bothered*) to 3 (*severely bothered*). The total score is in the range 0–60, and a participant thus had more severe insomnia when there score was higher. We considered a cutoff score of 20 to indicate clinical insomnia^28^. The test–retest reliability and Cronbach’s alpha of the PIRS_20 are 0.92 and 0.95, respectively^30^. Its Cronbach’s alpha is 0.94 in this presenting study.

#### Chen Internet addiction scale—gaming version

The self-report Chen Internet Addiction Scale (CIAS) evaluates the following aspects of Internet addiction: withdrawal, tolerance, time management, compulsive use, and problems with health and interpersonal relationships^31^. The scale’s 26 items are evaluated on a 4-point Likert scale ranging from 1 to 4. The CIAS—gaming version (CIAS-G) is a modified version of the CIAS that is used to determine to what degree individuals are addicted to online gaming. The Cronbach’s *α* of the CIAS-G is 0.96^32^, and the total score varies between 26 and 104. A participant was defined as having more severe IGD when they had a higher CIAS-G score.

After the psychiatrist conducted diagnostic interviews with the participants to identify adult ADHD, the participants completed the CIAS-G, PIRS_20, and CSM.

### Statistical analysis

Chi-square analysis was employed to evaluate the association between circadian typologies and IGD was analysed. Fisher's exact test is used when one or more expected numbers in the cell are less than 5. Analysis of variance and Tukey’s honestly significant difference post hoc comparison were used to compare the ages, CSM scores, PIRS_20 scores, and CIAS-G scores of the three groups.

To evaluate the associations of the CSM and PIRS_20 scores with IGD when controlling for sex and age, we employed forward regression analysis. Correlations between the CSM, PIRS_20, and CIAS-G scores were evaluated through Pearson’s analysis.

Independent-samples *t* tests were used to identify differences in the CSM and PIRS_20 scores between the IGD groups with and without ADHD.

### Ethics

The purposes and methods of this research were explained in detail to all the participants, and informed consent was then acquired from them. We obtained approval for the present study from Kaohsiung Medical University Hospital’s Institutional Review Board. All methods were performed in accordance with the relevant guidelines, regulations, and the Declaration of Helsinki. No participant in this study is a minor, whose informed consent must have been obtained from a parent and/or legal guardian.

## Results

The final analysis involved all 207 participants. Of these participants, 162 (78.3%) and 45 (21.7%) were men and women, respectively. Each of the three groups comprised 54 men and 15 women.

Analysis of variance revealed the IGD group to have the lowest CSM scores, followed by the regular gamer group and control group (*F* = 42.40, *p* < 0.001). These findings thus indicated that the IGD group had a stronger eveningness preference than the remaining participants. The IGD group was found to have significantly more severe insomnia than the remaining participants (*F* = 40.66, *p* < 0.001) as well as significantly more severe IGD than the regular gamer and control groups (*F* = 357.58, *p* < 0.001; Table 1).

**Table 1:** The difference in circadian typologies, insomnia, and severity of internet gaming disorder (IGD) within IGD group, regular gamers, and controls.

	IGD (N = 69) (N%)	Regular gamer (N = 69) (N%)	Controls (N = 69) (N%)	χ^2^ test	
Gender					
Women	15 (33.3%)	15 (33.3%)	15 (33.3%)	0.00	
Men	54 (33.3%)	54 (33.3%)	54 (33.3%)		
Circadian typologies					
Evening Type	15 (83.3%)	3 (16.7%)	0	29.28*** (Fisher’s Exact)	
Intermitent type	54 (30.6%)	61 (34.7%)	61 (34.7%)		
Morning type	0	5 (38.5)	8 (61.5%)		

	IGD (*n* = 69) Mean ± SD	Regular gamer (*n* = 69) Mean ± SD	Controls (*n* = 69) Mean ± SD	F value	Post Hoc (Cohen's *d*)
Age	25.32 ± 4.20	24.59 ± 3.41	26.87 ± 3.82	6.38**	Control > regular gamer (0.58)
Circadian typologies	27.26 ± 5.37	33.29 ± 6.22	36.20 ± 5.83	42.40***	Control > regular gamer (0.42) > IGD (0.88)
Insomnia	26.19 ± 10.70	15.22 ± 8.60	12.25 ± 9.27	40.66***	IGD > Control (1.24)
The severity of IGD	82.86 ± 10.49	55.87 ± 14.69	30.86 ± 8.11	357.58***	IGD > regular gamer (1.11) > C ontrol(1.04)

**p < .01; ***p < .001.

Circadian typologies: The score of the Composite scale of morningness; Insomnia: the score of Pisburg Insomnia Scale 20 items version; Severity of IGD: the score of Chen Internet Addiction Scale—gaming version.

Post Hoc analysis is evaluated by Tukey’s honestly significant difference test.

Forward logistic regression analysis of the associations of IGD (the IGD group vs. the control group) with circadian typologies and insomnia was performed (Table 2). The results indicated significant associations of CSM score (Wald *χ*^2^ = 19.73; *p* < 0.001; odds ratio (OR) = 0.84; 95% confidence interval (CI) [0.78, 0.91]) and PIRS_20 score (Wald *χ*^2^ = 16.44; *p* < 0.001; OR = 1.09; 95% CI [1.05, 1.13]) with IGD when controlling for age and sex. This result suggested that the participants with IGD tended to exhibit eveningness preference and insomnia.

**Table 2:** Forward logistic regression model for the association between the IGD group (versus the control group) and circadian typologies and insomnia after controlling for sex and age.

Variables	Wald	Odds ratio	95% CI
IGD group versus control group			
Age (year)	0.46	1.03	0.94–1.14
Gender	2.43	2.13	0.82–5.54
Circadian typologies	19.73***	0.84	0.78–0.91
Insomnia	16.44***	1.09	1.05–1.13

***p < .001.

Circadian typologies: The score of the Composite scale of morningness; Insomnia: the score of Pisburg Insomnia Scale 20 items version.

How circadian typologies, insomnia, and IGD severity were interrelated in the IGD group was analysed using Pearson’s correlation. In this group, CSM score exhibited a significantly negative correlation with PRIS_20 score (*r* =  − 0.38; *p* = 0.001), whereas IGD severity exhibited a positive correlation with sleep quality (*r* = 0.42; *p* = 0.001).

We thoroughly evaluated the differences in circadian typologies and insomnia severity between those in the IGD group with and without ADHD. The numbers of participants from the IGD group with and without ADHD were 35 and 34, respectively. The ADHD data are detailed in a previous report related to the comorbidities of IGD^33^.

The CSM and PRIS_20 scores were significantly lower (*t* = 2.32, *p* = 0.02, Cohen’s *d* = 0.56) and significantly higher (*t* = 2.51, *p* = 0.02, Cohen’s *d* = 0.60), respectively, among those with IGD and ADHD than among those with IGD but not ADHD. ADHD was thus concluded to have had exacerbating effects on circadian typologies and insomnia severity in the IGD group (Table 3).

**Table 3:** The difference in circadian typologies and insomnia between the IGD with ADHD and those without.

Variables	IGD with ADHD (N = 35)	IGD without ADHD (N = 34)	*t*-value (*p*)	Cohen’s *d*
	Mean ± SD	Mean ± SD		
Insomnia	29.26 ± 10.90	23.04 ± 9.67	2.51 (0.02)	0.60
Circadian typologies	25.83 ± 5.13	28.74 ± 5.29	− 2.32 (0.02)	0.56

*p < .05.

Circadian typologies: The score of the Composite scale of morningness; Insomnia: the score of Pisburg Insomnia Scale 20 items version.

## Discussion

The correlations among insomnia, circadian typologies, ADHD, and IGD were investigated in this study. We now describe our main findings. First, the IGD group experienced more severe insomnia than the regular gamer and control groups. Second, the participants with IGD exhibited the tendencies of eveningness preference and poor sleep quality. Third, ADHD exerted exacerbating effects on circadian typologies and insomnia severity in the IGD group.

### Relationships among circadian typologies, gaming behavior, and IGD

The regular gamer group exhibited a stronger eveningness preference than did the control group. In several cross-sectional studies, stronger eveningness preference has been discovered to be associated with more frequent Internet or social media use and gaming behavior^11,12,34^. The blue light emitted by mobile phones and computer screens block melatonin secretion and delay the phases of the circadian clock, thereby resulting in disruption of the circadian time structure^35^. A study suggested that exciting gaming affects sleep latency and rapid eye movement sleep^36^. We discovered that gaming behavior contributes to eveningness preference, providing further support to other studies’ findings.

The results also indicated that compared with the regular gamer group, the IGD group exhibited a stronger tendency of eveningness preference (Table 1). In a longitudinal study, Chen and Gau^13^ found that Internet addiction prospectively predicts disturbance in the circadian rhythm of adolescents and children^13^. In a systematic review, Kristensen et al.^37^ reported that problematic gaming has significant associations with nocturnal awakening^37^, the eveningness chronotype, and delayed sleep phase disorder. Furthermore, the circadian gene plays a role in addiction^8^. An animal study indicated that rats are more vulnerable to cocaine’s reinforcement effect at midnight than in the morning^38^. Thus, people who are prone to staying awake and playing video games at midnight could be more vulnerable to the reinforcing pleasure of gaming. We found that gaming behavior and addiction to gaming are associated with eveningness preference, again adding support for other findings.

### IGD–insomnia relationship

Individuals who exhibit Internet addiction or pathological Internet usage are more likely to have insomnia as well as to have daytime sleepiness and poor sleep quality^6,15^. Many cross-sectional studies have reported similar observations regarding IGD, suggesting an association of addictive gaming with sleep disturbances, including less time spent asleep and sleep quality deterioration^12,17,39^. In agreement with the aforementioned results, the present results indicate that individuals with IGD experience more severe insomnia than do those without IGD (Table 2). Moreover, IGD severity is positively correlated with insomnia (Table 3). This finding is consistent with the evidence that higher IGD severity is associated with higher insomnia severity^4^. Sleep displacement may be the mechanism underlying the IGD– insomnia association. IGD may cause a reduction in sleep duration, daytime sleepiness, or sleep deprivation^39^, all of which subsequently lead to insomnia and desynchronization^35^. The duration of playing a video game is negatively correlated with sleep quality^5^. In addition, exposure to bright light before sleep causes the production of melatonin to be suppressed in the human body, which can contribute to delayed sleep onset^40^. Thus, excessive engagement in Internet gaming may lead to insomnia.

On the other hand, psychiatric comorbidity, such as depressive or anxiety disorder, is associated with GD^33^. For example, problematic gaming has been reported to associate with depression and stress^41^. Further, both stress^42^ and depression^43^ could contribute to insomnia. Thus, psychiatric comorbidity might play an interplay role between GD and insomnia and should be an important issue in future studies to understand the association between GD and insomnia.

### Effects of ADHD on the circadian typologies and insomnia severity of those with IGD

According to the results presented herein, ADHD has exacerbating effects on circadian typologies and insomnia in those with IGD. Systematic reviews confirmed that IGD is positively correlated with the inattention subscale of the ADHD scale^19^ or hyperactivity symptoms^20^. Individuals with ADHD have been discovered to exhibit a shorter sleep time, a later chronotype or delayed circadian rhythm, and more frequent circadian and sleep disorders compared with those without ADHD^23,24,44^. A study found that in a subgroup of patients with ADHD, circadian rhythm played a causative role in issues with sleep^23,25^. Online gaming has been associated in one previous study with volume changes in the ventral striatum, which is a crucial brain region that implements the brain’s reward mechanisms, as well as with changes in brain regions that have been found to participate in impulse control, sensory-motor coordination, attention and control, emotional regulation, and motor functions^45^. Online gaming was suggested by Park et al.^21^ to possibly be a method of self-medication for patients with IGD and attentional problems^21^. The complex sleep–ADHD relationship may be related to neuroanatomical and functional overlap between regions of the brain that participate in sleep regulation, arousal, and attention^46^. In addition, ADHD and delayed circadian rhythm may be associated with genetics and have a shared etiology^24^. Because ADHD exerts exacerbating effects on circadian typologies and insomnia severity in those with IGD, the sleep problems and ADHD symptoms of adults with IGD should be carefully evaluated in clinical settings.

### Limitations

Regarding this study’s limitations, first, inadequate statistical power was achieved because of the small sample, which was caused by the strict inclusion criteria. Second, only the data from diagnostic interviews and questionnaire surveys were analysed. The use of questionnaires may have resulted in the severity of various factors being underestimated. Third, this study involved cross-sectional research; thus, understanding causes and effects was difficult. Last, the severity of IGD is assessed by the CIAS-gaming version, but not by international scales, such as Internet Gaming Disorder Test (IGDT-10)^47^, Internet Gaming Disorder Scale-Short Form (IGDS9-SF)^48^, and Gaming Disorder Test (GDT)^49^. Evaluating this presenting topic by using an international scale might contribute to the comparison between different regions over the world.

## Conclusion

In this study, we discovered that adults with IGD were more likely to experience eveningness and insomnia compared with regular gamers and the controls. Moreover, ADHD was found to exert exacerbating effects on circadian typologies and insomnia severity in those with IGD. If effective interventions are to be designed for individuals with IGD, scholars should investigate this disorder from various perspectives. Strategies implemented for early IGD intervention are critical for improving sleep problems. Future studies can adopt different perspectives to overcome the limitations of the present study, such as casual relationships, and thus discover directions for IGD interventions.

## Data Availability

The datasets used and/or analysed during the current study available from the corresponding author on reasonable request.
